# The Mystery of a Unilateral Headache Ultimately Diagnosed as Moyamoya Disease

**DOI:** 10.7759/cureus.26816

**Published:** 2022-07-13

**Authors:** Mercedes Malone, David Ritchie

**Affiliations:** 1 Internal Medicine, Hospital Corporation of America (HCA) Florida North Florida Hospital, Gainesville, USA; 2 Surgery, Hospital Corporation of America (HCA) Florida Kendall Regional, Miami, USA

**Keywords:** encephaloduroarteriosynangiosis (edas), edas, encephaloduroarteriosynangiosis, headache, generalized headache, case report, moyamoya disease

## Abstract

Moyamoya disease (MMD) is a rare chronic cerebrovascular occlusive disease characterized by progressive stenosis or occlusion of the intracranial internal carotid arteries and their proximal branches, with subsequent abnormally formed collateral vessels. Moyamoya disease is typically found in children of East Asian descent and is the most common pediatric cerebrovascular pathology in East Asian populations. However, moyamoya disease can be present without any predisposing factors, and this is what distinguishes the disease from the syndrome. Moyamoya syndrome is typically associated with other conditions such as sickle cell disease or neurofibromatosis. The syndrome can also be seen in patients who have had exposure to cervical or neck radiation.

We present a rare case of an adult Peruvian woman who initially presented with a severe right hemispherical headache, which was ultimately diagnosed as MMD. This report and the discussion aim to provide more understanding of moyamoya disease and how it can be incidentally discovered in an unsuspected patient without any predisposing factors. The fact that the patient lacked any predisposing factors makes moyamoya disease, and not the syndrome, the diagnosis. Currently available treatments are limited. One specialized therapeutic approach is a procedure called encephaloduroarteriosynangiosis (EDAS), which aims to involve the transposition of a segment of a scalp artery onto the surface of the brain to permit the additional formation of collateral arteries.

We aim to highlight the management and treatment of a case of moyamoya presenting as a severe right hemispherical headache in a patient without any predisposing factors.

## Introduction

Moyamoya is a Japanese term that translates to "puff of smoke." The medical analogy stems from the appearance of cerebral angiography of abnormal vascular collaterals that resemble diaphanous clouds. These collaterals develop adjacent to the stenotic vessels. The presentation of moyamoya disease (MMD) is highly variable and can occur at any age and can present in various ways, including headaches, seizures, cognitive impairment, transient ischemic attacks, or cerebral vascular accidents [1,2]. The symptoms are manifestations of cerebral ischemia or intracranial hemorrhage. The etiology of Moyamoya disease is not fully understood. However, there appears to be a genetic component to the disease as it is more prevalent in patients of East Asian heritage such as Japanese and Koreans [3,4]. The condition is typically diagnosed after angiography or magnetic imaging of arteries illustrate findings of stenosis occluded at the terminal portions of the internal carotid artery [5]. Currently, there is no specific treatment to prevent or reverse MMD progression, and therapeutic strategies include both medical and surgical interventions. A surgical procedure known as encephaloduroarteriosynangiosis (EDAS) has been found helpful over time in allowing the formation of new collaterals in the brain for those with MMD [6].

## Case presentation

A 51-year-old Peruvian female with a past medical history significant for migraines and Meniere's disease that had resulted in chronic right-sided hearing loss presented to our institution with a primary complaint of severe headache accompanied by nausea, confusion, and lethargy. Her symptoms initially began two days prior to her presentation when she attended a social gathering and consumed numerous alcoholic beverages. Her headache persisted and worsened after a day of resting with her traditional therapeutic regimen of meditation and over-the-counter anti-inflammatory medications. Due to the increase in pain, she decided to seek medical evaluation.

**Figure 1:**
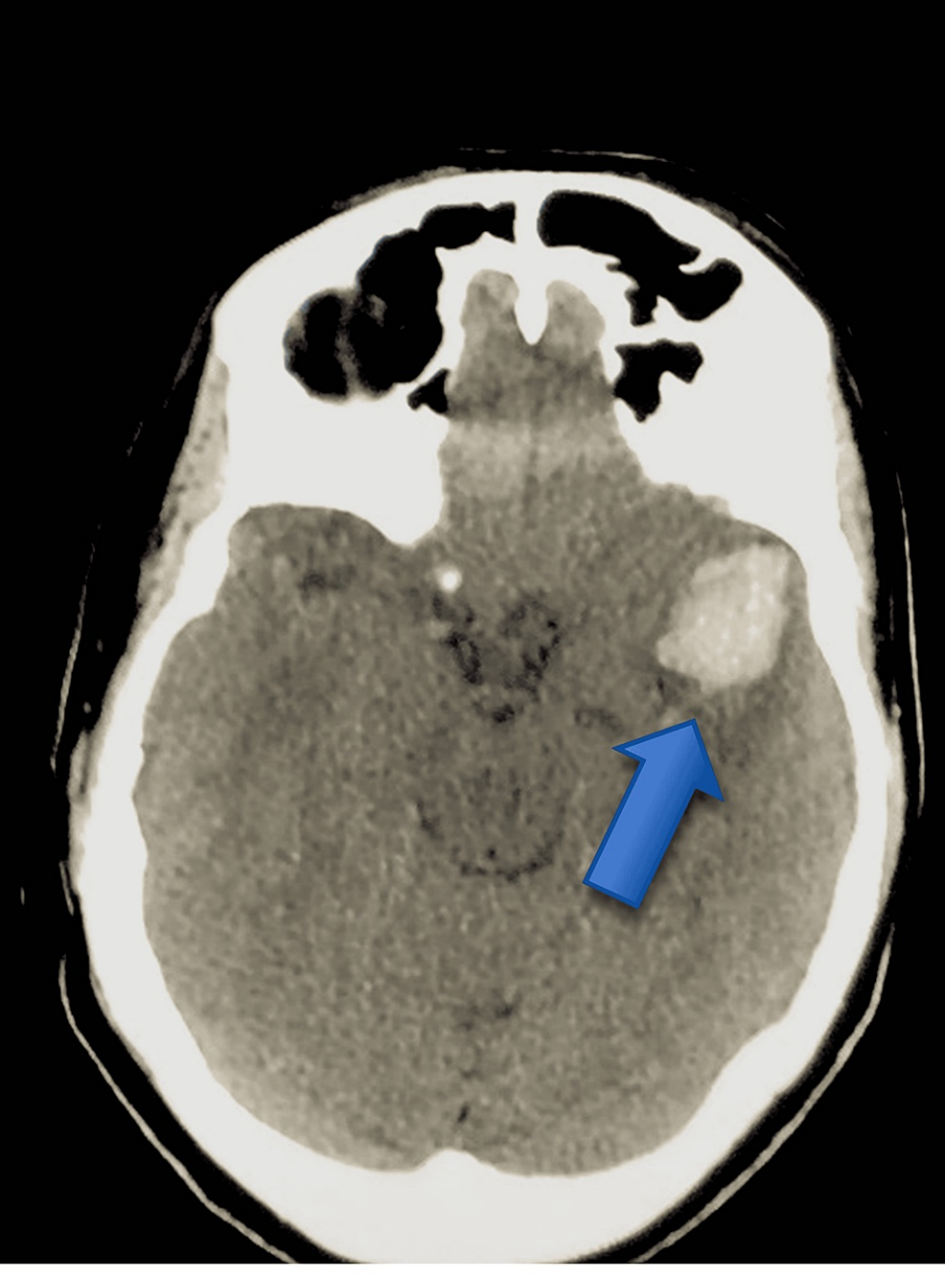
CT image of intraventricular hemorrhage in the left lateral ventricle. The arrow points to the 2.8 cm × 2.1 cm × 3.2 cm left temporal lobe hemorrhage with intraventricular blood in the left lateral ventricle and possibly temporal horn on the left and surrounding edema.

**Figure 2:**
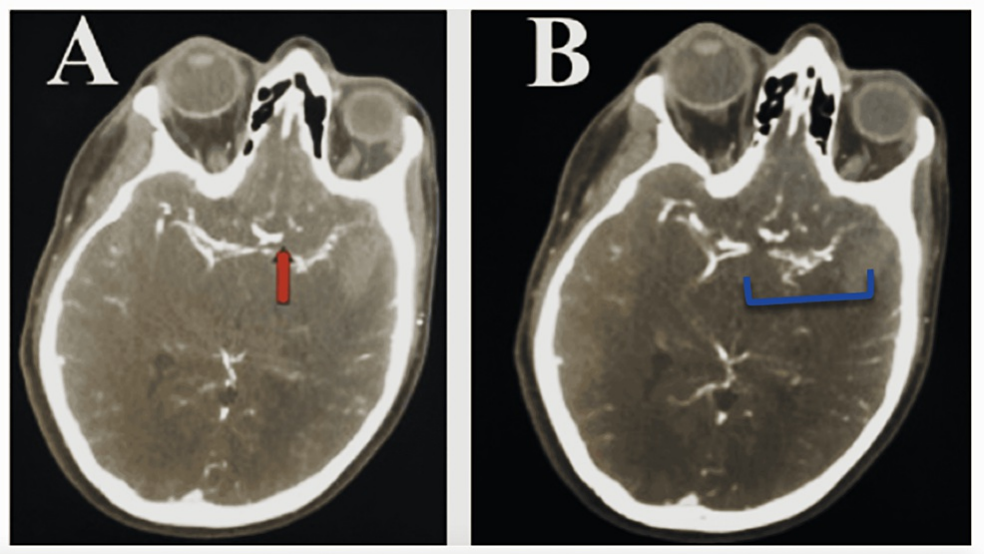
CTA of head and neck showing stenosis and abnormal vascular collaterals. (A) There is an abnormal appearance of the supraclinoid left internal carotid artery at the bifurcation. The arrow points to an area of irregular stenosis along the entirety of the pre-trifurcation of the left middle cerebral artery. (B) The blue bracket encloses the area where there are multiple small vessels arising from this segment of the middle cerebral artery suggesting MMD. CTA: computerized tomographic angiography.

On arrival, she was hemodynamically stable and afebrile, with vital signs that were all within normal limits. The only abnormal laboratory value was mild hypokalemia, which resolved after appropriate potassium replenishment. Due to the severity of her pain, a computed tomography (CT) of the brain without contrast and CT angiography head and neck were completed. They revealed a left temporal lobe hemorrhage with left-sided intraventricular hemorrhage (Figure 1) and high-grade stenosis in a long segment of the left middle cerebral artery (MCA) close to the trifurcation of the internal carotid artery (Figure 2). These radiological imaging findings are highly consistent with the presence of moyamoya disease.

**Figure 3:**
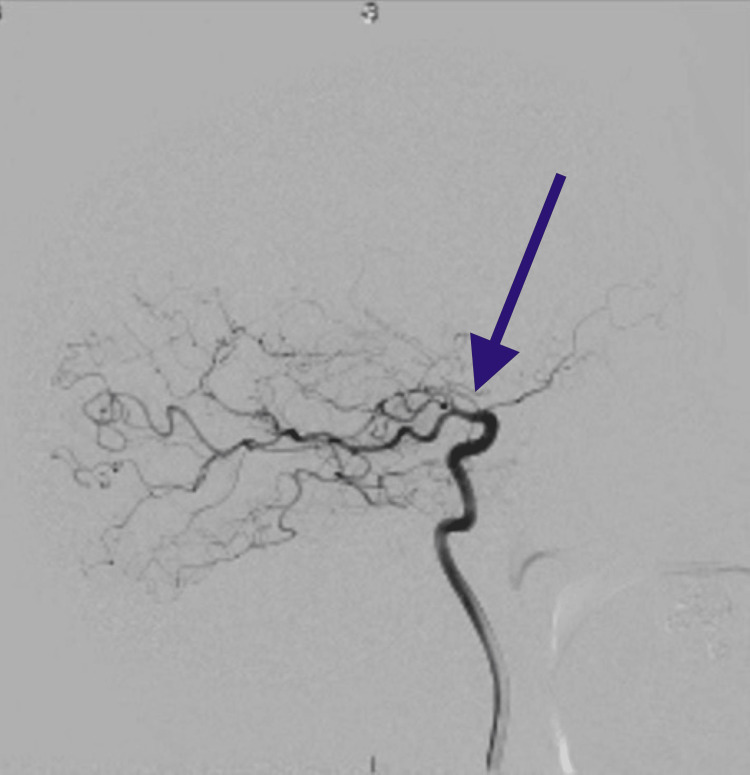
Cerebral angiogram. This is an angiogram that illustrates occlusion of the supraclinoid segment of the left internal carotid artery. The arrow points to the area of stenosis and the subsequent vessels have the “smoke-like” appearance of moyamoya disease.

Neurosurgery was consulted and evaluated the patient. She underwent a cerebral angiogram that revealed occlusion of the supraclinoid segment of the left intracranial artery (Figure 3). This finding was believed to be related to MMD. The patient was placed on anti-epileptic medication for seizure prophylaxis and underwent a successful procedure called EDAS due to evidence of cerebral ischemia. Due to her intraventricular hemorrhage, antiplatelet medication was not used.

Direct and indirect revascularization are two different revascularization methods that can be performed. In certain circumstances, it is possible to perform a combination of both methods. The decision as to whether to perform direct or indirect vascularization is based upon the patient's age and the size or fragility of the recipient artery. The ultimate goal of both procedures is to reduce the risk of future strokes or ischemia. Direct revascularization is a surgical procedure that is more difficult to perform but is preferred for young children and involves connecting the superficial temporal artery to the MCA and creating a bypass. Direct is the preferred revascularization method given that the effects are more immediate in comparison to indirect. The procedure of direct revascularization is limited if the recipient artery is fragile or small [6,7].

Indirect revascularization is an interventional technique that focuses on developing a new arterial inflow into an area that is not being oxygenated. Indirect bypass requires more time, sometimes roughly three to six months, to create new collateral and experience the full effects. The patient’s own body stimulates angiogenesis and creates collaterals from the transposed vessel that connect to the collateral vessels already formed in MMD. This technique enhances nutrient and oxygen-rich blood to reach the brain. EDAS is an indirect revascularization method given that it does not involve directly connecting the superficial temporal artery to the middle cerebral artery. In EDAS, a scalp artery is transected, mobilized, and then placed on the surface of the brain. Hypoxia then stimulates the formation of new collaterals that grow and connect with previously formed collaterals. This transposition of a segment of a scalp artery onto the surface of the brain bypasses the occluded segment [7,8].

## Discussion

Headache is a common chief complaint of patients who present to the emergency department and the list of differential diagnosis can include traumatic brain injury, cerebral vascular accident, or possibly seizure. MMD is a rare entity and oftentimes missed, frequently presenting in the third or fourth decade of life. The disease is mostly geographically concentrated in Asia and less commonly found in the United States. One epidemiological study in the United States used nationwide inpatient data and estimated the prevalence of the disease in three years from 2005 to 2008 to be roughly around 7,473 [9].

This report brings awareness of this rare disease and the importance of early recognition and management. Moyamoya disease should be included in the differential diagnosis for patients who present to the ED with headaches and characteristic CT findings. A delay in diagnosis and treatment can lead to significant morbidity and mortality for the patient. It is important to perform imaging in individuals with unusual headache features like persistence despite usual therapy.

A headache can be a common initial manifestation of moyamoya disease, and one study even describes these headaches as a harbinger or a presenting sign due to the inflammatory action of chemicals around the cerebral blood vessels [8]. MMD is described as having a genetic component, usually found in those with Asian ancestry. It is uncommonly found in adult patients of Latin American descent, as in a reported patient who was originally born in Peru and lacked known Asian ancestry. The patient in our case was otherwise healthy except for her history of migraine headaches and Meniere’s disease. Prior to her presentation to our hospital, she had consumed alcoholic beverages and, with her history of migraines, her symptoms could have easily been dismissed as having another migraine and the condition would have been misdiagnosed and mismanaged, leading to a delay in diagnosis and possible morbidity or mortality. Alarm signs where imaging is indicated include headache with fever, history of malignancy, neurologic deficit, new-onset age over 50, new headache pattern, papilledema, and post-traumatic. In this case, the new headache pattern was a clue that warranted further evaluation.

Treatment for moyamoya includes acute management and secondary prevention. Acute management is mostly symptomatic with antiplatelet therapy such as aspirin. Revascularization is sometimes necessary when patients present with cerebral ischemia. Direct or indirect revascularization are both options; in this case, indirect EDAS was used. Secondary prevention also includes aspirin or cilostazol. The condition is typically diagnosed after angiography or magnetic imaging of arteries illustrate findings of stenosis-occluded at the terminal portions of the internal carotid artery [5]. Although bilateral internal carotid vessels are typically affected, in this case the patient has a unilateral stenotic occlusion of the left terminal portion of her internal carotid artery.

## Conclusions

Headache is a common presenting symptom in the adult population, and a nuanced approach is necessary to identify patients who require more than symptomatic treatment. The case presented here illustrates a woman in her 50s who presented with a headache refractory to her regular medication in the setting of a history of migraines. A broad differential including intracerebral hemorrhage and vascular abnormalities prompted further imaging, which revealed vascular findings consistent with moyamoya disease. By discovering this condition, appropriate treatment and prevention were offered, which included indirect revascularization and antiplatelet therapy.
